# Comparison of Two Old Phytochemicals versus Two Newly Researched Plant-Derived Compounds: Potential for Brain and Other Relevant Ailments

**DOI:** 10.1155/2014/682717

**Published:** 2014-05-08

**Authors:** Chun-Mei Wang, Willmann Liang, D. T. Yew

**Affiliations:** ^1^Brain Research Center, Institute of Chinese Medicine, The Chinese University of Hong Kong, Shatin, New Territories, Hong Kong; ^2^Brain Research Center, School of Biomedical Sciences, Faculty of Medicine, The Chinese University of Hong Kong, Shatin, New Territories, Hong Kong

## Abstract

Among hundreds of formulae of Chinese herbal prescriptions and recently extracted active components from the herbs, some of which had demonstrated their functions on nervous system. For the last decade or more, *Gingko biloba* and *Polygala tenuifolia* were widely studied for their beneficial effects against damage to the brain. Two compounds extracted from *Apium graveolens* and *Rhizoma coptidis*, butylphthalide and berberine, respectively, received much attention recently as potential neuroprotective agents. In this review, the two traditionally used herbs and the two relatively new compounds will be discussed with regard to their potential advantages in alleviating brain and other relevant ailments.

## 1. Introduction

In a study of over hundreds of formulae of Chinese herbal prescriptions for central nervous system (CNS) ailments in the last fifty years, we had found* Polygala tenuifolia* as one of the leading herbs which appeared in at least 40% of these formulae. In the recent thirty years, however, there were other newly studied herbs centered around the CNS which came into view and these were* Ginkgo biloba*, butylphthalide, and berberine, the last two compounds most recently isolated from* Apium graveolens* and* Rhizoma coptidis*, respectively. The more traditionally used Gingko and Polygala share common properties such as antioxidation, antiapoptosis, and neuroprotection in the context of Alzheimer's disease. While the newer compounds also elicit many if not all of these effects, they may have potential values in other conditions such as affective disorders, diabetes, hypertension, and stroke. In this concise review, we will highlight the findings associated with each of these agents and comment on potential usage as therapeutics for brain and other diseases.

## 2. *Ginkgo biloba*

**Figure 1:**
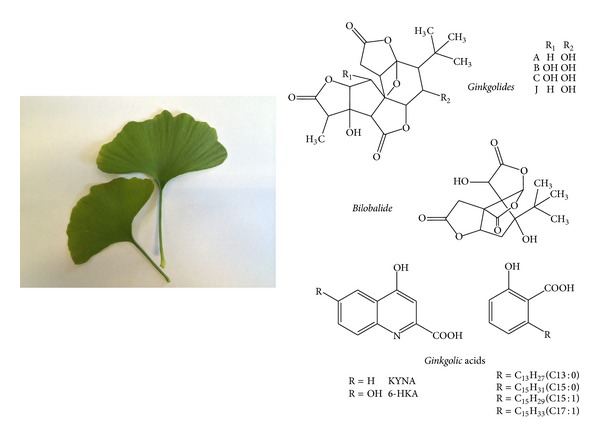
Leaf of* Ginkgo biloba* and chemical structure of some of the important constituents of EGb 761 [6].

*Ginkgo biloba* had been manufactured as an extract under the well-known formulation of EGb 761 (Figure 1). In experimental culture studies, when the survival of rat pheochromocytoma cells was compared with and without treatment of* Ginkgo biloba*, and during the addition of a serum, serum deprivation or reperfusion of serum (which induced oxidative damages), results indicated that* Ginkgo biloba* imparted neuroprotection and prevented cell death which worked collaboratively with addition of serum. The logical suggestion was, therefore, the active ingredients in EGb 761 could work with trophic factors in the blood [1]. Along the same line of experiments, H_2_O_2_ could induce cell death in SH-SY5Y, a neuroblastoma cell line. Associated damage included DNA fragmentation, damage of mitochondrial membrane, and activation of intermediate early genes and kinases and subsequently caspases [2]. H_2_O_2_ could as well lead to decrease of cellular glutathione (GSH) while EGb 761 could reverse this trend. The summarized picture of this damage appeared to begin with hyperoxidation leading to membrane changes followed by activation of signaling pathways and finally nuclear fragmentation in the* in vitro* studies. In the* in vivo* studies, old (40 weeks old) accelerated aging mice had been employed, and the effect of EGb 761 was studied on the mitochondria of platelets and hippocampi of this strain. During aging, there was an obvious downregulation of cytochrome oxidase, ATP, and GSH activities in the mitochondria and EGb 761 appeared to protect against all these downfalls [3]. EGb 761 was found to be able to cross the blood brain barrier with ease in old and postmenopausal animals [3, 4]. In the gerbil model induced with global ischemia of the brain and which was subjected to subsequent reperfusion, mitochondria cytochrome c-oxidase (COX) downregulation could be amended by pretreatment with oral administration of EGb 761 (up to 100 mg/kg) or its constituent bilobalide for 7 days before injury [5].

It is however unclear whether EGb 761 could alleviate and protect the cell from all these adverse events or act only during some critical periods of the events. In addition, it was noted in these experiments that very high dosage of pure ginkgo (EGb 761) might have severe side effects over the routine dosage [2]. On the other hand, bilobalide in EGb 761 had been documented to provide protection even after withdrawal of the drug against amyloid beta (A*β*) formation in abnormal aging, H_2_O_2_, and serum deprived apoptosis and it was likely that it mainly acted through the PI3K/Akt pathway [7]. Equally important, EGb 761 had the experimental ability to block A*β*1-42 cytotoxicity in SH-SY5Y cells, possibly due to its other components of quercetin and ginkgolide B in this study, and these two components were antioxidants and platelet aggregation factor antagonists [8].

In essence, flavonoid and ginkgolides in ginkgo were specifically antioxidants which prevented lipid peroxidation. As well, these components also regulated glucose while bilobalide could increase ATP in tissues via its action on the respiratory chain and cytochrome oxidase [9]. During cerebral ischemia in the experimental animals, bilobalide and ginkgolide B pretreatment would reduce infarct volume and rendered cells resistant to neurotoxicity. Ginkgolides B and J together had been shown as well to protect cells in culture [10].

In the human subjects, it was known for at least ten years that* Ginkgo biloba* improved cognitive performance, possibly by acting as an acetylcholinesterase inhibitor [11]. This drug worked better than donepezil and associated acetylcholinesterase (AChE) inhibitors and was better tolerated for patients with mild and moderate dementia [12–14]. These research studies correlated well patient studies on the Alzheimer's Disease Assessment scale (ADAS-Cog), which presented a 30% increase of scores after 6 months of Ginkgo treatment [13]. Similar improvements in cognitive functions were also recorded by the Syndrom-Kurztest [15]. With longer time of drug treatment in patients, for example, 52 weeks, additional improvements were recorded by ADAS-Cog and Geriatric Evaluation by Relative's Rating Instrument (GERRI). For the patients with severe dementia, the improvement after Ginkgo treatment was, however, limited, though it appeared to be able to control further deterioration [16, 17].

Nitric oxide (NO), another known positive molecule producing radicals in the Alzheimer's brain [18], could be produced* in vitro* by treating hippocampal cells in culture with sodium nitroprusside and this event could be reversed by EGb 761 or its flavonoid fraction (CP 205) whilst other major constituents like bilobalide or ginkgolide B had no effect as they would not inhibit NO induced protein kinase C activities [19].

## 3. *Polygala tenuifolia*

**Figure 2:**
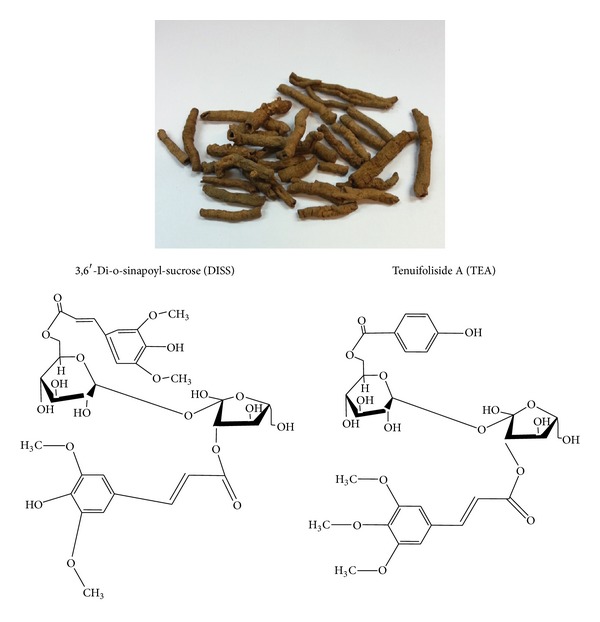
The picture of* Polygala tenuifolia* and the structure of its two components, DISS and TEA [20].

One of the Chinese herbal agents used for the CNS for centuries was the root of Radix* Polygalae*. There were many species of the Polygalae genus, the most common one being* Polygala tenuifolia*. For most of the studies in the literature, ethanol and methanol extraction were employed. The difference between these and the simple extraction by water remains to be elucidated.* Polygala tenuifolia* is among the most frequently used herb in 3000 years of history of Chinese medicine for the treatment of neuronal problems [21]. In this drug, four of the important constituents, oligosaccharide 3,6′-di-o-sinapoyl-sucrose (DISS) [20], tenuifoliside A and tenuifoliside B (TEA, TEB) [20, 22], and 3,4,5-trimethoxycinnamic acid (TMCA) [23] were identified as biomarkers (Figure 2).

In culture of neuroblastoma cells, DISS had been found to protect the SH-SY5Y cells from glutamate induced apoptosis [24]. Other studies indicated many saponins in this herb could protect cells from serum deprived injury [25]. TMCA in this herb could upregulate pentobarbital-induced sleep [23]. In models which had corticotrophin releasing hormone induced stress, TMCA appeared to be able to enhance sleep induced by pentobarbital in these stressed rats [26]. These enhanced episodes of sleep were related to activation of glutamic acid decarboxylase (GAD) and gamma-subunit of GABA alpha receptors [23]. Mixture of this herb with* Ginseng *likely increased neurotransmitters and neurotrophins [27]. In neuronal cultures administrated with 6-hydroxydopamine (6-OHDA) and in 1-mehtyl-4-phenyl-1,2,3,6-tetrahydropyridine (MPTP) treated mice to induce Parkinson's disease, the dopaminergic neurons gained protection from actions of reactive oxygen species, nitric acid production, and increased caspase 3 activities by administration of* Polygala tenuifolia* [28]. In the hippocampus of the rat, this herb promoted neurite outgrowth from precursor cells after injury [29]. In the aged mice which were tested with passive avoidance test or Y type maze, tenuifolin improved latency time and reduced error via the increased levels of norepinephrine (NE) and dopamine (DA) and decreased AchE [30]. Spatial cognition in an eight-arm radial maze was also noted to be improved in mice after* Polygala tenuifolia* treatment [31]. In elderly human,* Polygala tenuifolia* was used as a prophylactic and the Consortium to Establish a Registry for Alzheimer's Disease Assessment Packet (CERAD) and Mini-Mental State Examination (MMSE) tests were used in this cohort and illustrated an improvement after* Polygala tenuifolia* administration [32]. It is also interesting that this drug in the experimental animals did not only act on the cerebral cortex or specifically the hippocampus, but also majorly activated the basal forebrain which sent diffuse projections onto the cortex [33].

Major degenerations or stroke could be focal or global. In the latter case, the great quantity of debris might potentiate infection and the extent of damage would likely cause a blood brain barrier breakdown. Infection which normally did not occur might become imminent and inflammation could go along with infection.* Radix polygala* would inhibit NO production induced by lipopolysaccharide [34] as well as prevent lipid peroxidation [35] but was not documented for treating any associated infection.

A majority of the effects elicited by* Polygala tenuifolia* are shared by* Gingko biloba*. These include protection against NO-mediated oxidative damage, inhibition of apoptotic cell death, and modulation of cholinergic transmission. Another effect probably unique to* Polygala tenuifolia *may owe to its ability to increase NE level [30], which implicates its antidepressant properties. Indeed,* Polygala tenuifolia* might act as an inhibitor of NE reuptake to exert antidepressant effects in animals subject to tail suspension tests and forced swim tests [20, 36].

## 4. DL-3-n-Butylphthalide

**Figure 3:**
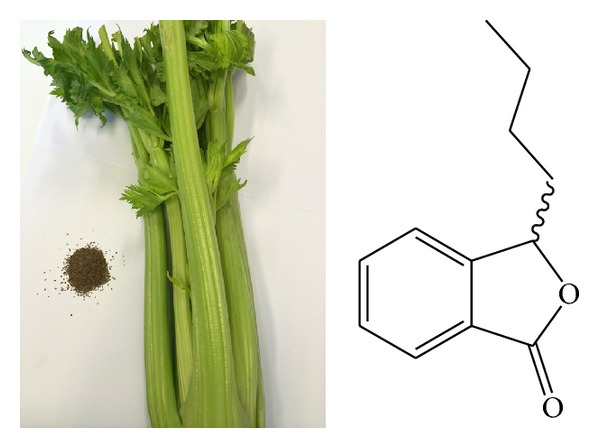
The pictures of* Apium graveolens* and its seeds and the structure of NBP [37].

DL-3-n-Butylphthalide (NBP) was a synthetic compound based on a single chemical L-3-n-butylphthalide isolated from seeds of* Apium*, usually* Apium graveolens* (Figure 3). In an experimental model where the spontaneously hypertensive (SHR) rat had occlusion of the middle cerebral artery to induce global stroke, pretreatment for 7 days and posttreatment for two weeks of this drug reduced infarct volume and lowered neurological deficit scores while, in the normotensive Wistar-Kyoto (WKY) rats, only posttreatment was found beneficial to the experimental stroke while pretreatment had no effect. It seemed therefore this agent also had an influence on the blood vessel or blood pressure control before stroke in the hypertensive animals [38] and thus improved its prognosis after stroke [39]. In poststroke patients, NBP improved the scores on Barthel index and Rankin scale [40]. Other cell and animal studies further indicated that NBP improved spatial memory via maze test, protected neuronal survival via blocking caspases, and enhanced angiogenesis involving vascular endothelial growth factor and the extracellular signal-regulated kinase 1/2-mediated pathways [41, 42]. A number of inflammatory mediators involved in the pathophysiology of astrocyte and microglia were suppressed as well by NBP. Proliferation of astrocytes was inhibited, together with decrease in cyclo-oxygenase-2, NF*κ*B, I*κ*Ba, TNF*α*, and IL-6 [43, 44]. It was demonstrated in SOD1-G93A mice (a model for amyotrophic lateral sclerosis) that activation of microglia and astrocytes, measured by CD11 and glial fibrillary acidic protein immunoreactivity, respectively, was lowered with NBP present [45].

NBP had been investigated for its effect on neurodegeneration and cell death. In double transgenic AD mouse (A*β*PP/PS1), NBP reduced A*β* production [46] while, in triple transgenic mouse (3xTg-AD), NBP intervened with APP processing and alleviated oxidative stress, implicating its potential use in Alzheimer's disease and other forms of dementia [47]. Similar results were obtained from rats subject to chronic cerebral hypoperfusion, with lower levels of the amyloid precursor protein and matrix metalloproteinase-2 after NBP treatment [48]. NBP prolonged life expectancy in the SOD1-G93A mice also, in addition to its immunomodulatory effects in the CNS mentioned above [49]. A derivative of NBP, ZJM 289, inhibited apoptotic cell death by suppressing the release of cytochrome c and translocation of apoptosis-inducing factor into the nucleus [50].

In addition to promoting angiogenesis, NBP and related derivatives also elicited other cardiovascular effects. In corticoid-induced hypertensive rats, blood pressure was markedly reduced by NBP [51]. Whereas neurological performance after ischaemic stroke was improved by NBP, its close derivative 6-amino-NBP might have an additional benefit by preventing activation and aggregation of platelets [52].

## 5. Berberine

**Figure 4:**
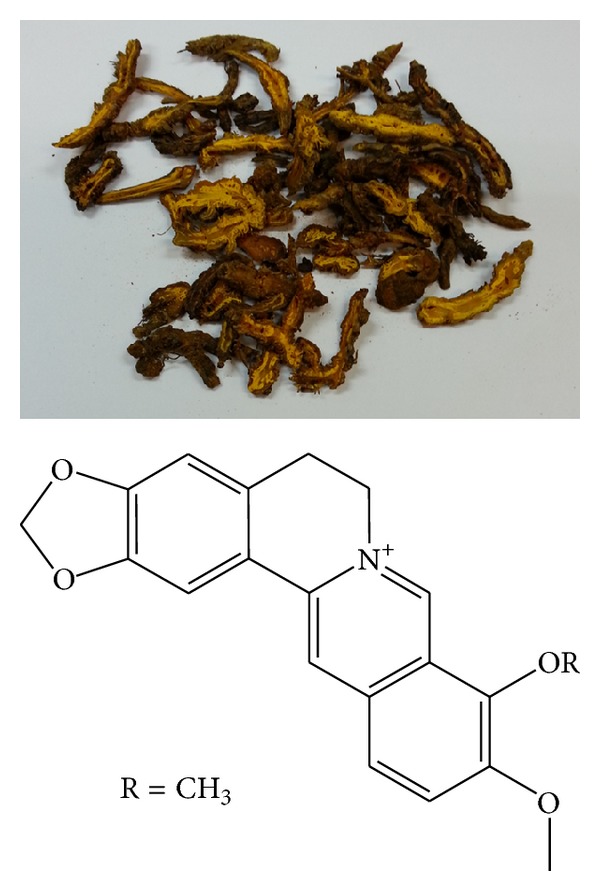
The picture of* Rhizoma coptidis* and the structure of berberine [53].

Berberine (Figure 4), an isoquinoline alkaloid, had been used for the last 30 years as a broad antibacterial agent for various types of infections, particularly for the gastrointestinal system. Recently this drug was shown to have other effects including anti-inflammation, cardioprotection, antitumor, antioxidative, and CNS protection [54]. In the peripheral nervous system, after injury of the rat sciatic nerve, accelerated axonal remyelination was observed with berberine treatment which included induction of neurite extension and differentiation in human neuroblastoma cells [55] and PC12 cells [56]. In retinoblastoma cells after oxygen deprivation, berberine improved cell survival [57]. Neurotoxin injection of MK801, a noncompetitive antagonist of NMDA receptor, would kill brain cells in neonatal rat, the effect which could be arrested at least in part by berberine treatment [58]. Berberine also induced expression of heme oxygenase-1 (HO-1) which was a potent antioxidant of superoxide production in astrocytes [59]. Berberine could further counteract other antioxidant enzymes like superoxide dismutase (SOD) and inhibited lactic dehydrogenase (LDH) release [60], while suppressed neuroinflammatory response via protein kinase activation and mitochondrial activation in microglia culture [61, 62]. Hyperoxidation induced by NO via nitric oxide synthase in the CNS of the rat could induce spatial memory impairment and the expression of interleukin-1 (IL-1) beta and these events could be ameliorated by feeding the experimental rat berberine at 50 mg/kg for 14 days [63]. Further immunosuppressant effects were demonstrated as there was less lymphocyte proliferation, coupled with lower level of TNF*α* and IL-10 [64]. The antioxidant and anti-inflammatory effects of berberine were reported in another study [65] with data also showing that oral bioavailability of berberine was improved when given together with the Ca^2+^ channel blocker verapamil. This finding is potentially important in clinical settings where patients are also being treated for cardiac dysrhythmia.

Potential usage of berberine in neurodegenerative diseases was suggested. Cholinesterase activity in type 1 diabetic rats was decreased and cognitive improvement was observed after berberine treatment [66]. In 1-methyl-4-phenyl-1,2,3,6-tetrahydropyridine- (MPTP-) treated dopaminergic neurons, neuronal cell damage was diminished after berberine treatment [67]. Motor activity was also improved, as was short-term memory [67]. This last finding could serve as additional evidence that berberine might be useful in Alzheimer's disease as well, which was supported by data showing that berberine downregulated the production of A*β* [68].

Unlike the three other herbs and herb-derived compound discussed earlier, a role for berberine in various affective disorders has been suggested as well. In animals subject to tail-suspension test and forced-swim test, berberine enhanced the effects of common antidepressants [69, 70]. The antidepressant properties of berberine might be due to its ability to increase the levels of monoamine transmitters by inhibiting their reuptake into the presynaptic terminal [71]. Also located at the presynaptic terminal are adrenergic *α*_2_ receptors, which elicit autoinhibition on NE release. The antidepressant effect of berberine, attributed to increased NE level in the synaptic cleft, could partially owe to its antagonistic property on presynaptic *α*_2_ receptors as well since the effect was enhanced by *α*_2_ receptor antagonist yohimbine [69].

The antipsychotic and anxiolytic effects of berberine were also related to monoamine transmitters. The potential use in schizophrenia was suggested when berberine was found to act as a D_2_-receptor antagonist [72], although it was also noted that dopamine level was increased in the brain as partly responsible for its antidepressant mechanism [71]. It is unclear if these influences on dopamine level and action may counter each other, diminishing the proposed antipsychotic effect. Future studies may also elucidate whether berberine will exacerbate extrapyramidal motor symptoms due to the blockade of D_2_ receptors. On the other hand, it was proposed that the anxiolytic effect of berberine resulted from its antagonism at 5-HT_2_ receptor [73]. This finding may indicate less severe motor side effects, if there are any, when berberine is used as an antipsychotic since atypical antipsychotics also act via 5-HT_2_ receptor blockade. A possible advantage of berberine over other antipsychotics is its ability to inhibit prolyl oligopeptidases, the activity of which is elevated in psychosis and not targeted by antipsychotics at present [74].

Elevated levels of prolyl oligopeptidases are present in diabetes as well, and inhibition of these enzymes may contribute to the antidiabetic effect of berberine. Berberine induced hypoglycemia in streptozotocin-induced diabetes in rats [66, 75]. In type 2 diabetic patients, berberine also demonstrated hypoglycemic effect, which was further enhanced by another herb* Silybum marianum* [76]. The relationship between hypoglycemia, protein metabolism, and prolyl oligopeptidase activity in diabetes has yet to be elucidated, after which the antidiabetic properties of berberine can be better understood.

## 6. Summary

In our view, all the above agents are useful as basic treatment against neurodegeneration. However, during episodes with massive bleeding (e.g., stroke) where infection appeared to be a likely consequence, the addition of an agent like berberine which has both protective effect and anti-infective effects on the CNS would be another important therapeutic approach in the treatment of CNS injury with traumatic episodes.

In fact, all of the four herbs (extracts) specified here in some ways can protect neurons from damage.* Ginkgo biloba *and* Polygala tenuifolia* are useful as ingredients to promote neuronal health and act as defender in superoxidation and against kinase formation as well as blockers of apoptotic pathway.* Polygala tenuifolia* apart from maintaining CNS health is also employed as sedative, hypnotic, or tranquillizer. The function of this herb can be compared and combined with other sedative agents like* Ramulus Uncariae cum Uncis* and* Semen Ziziphi Spinosae* which are also well known Chinese herbal sedative agents. NBP is better for treatment and prevention of cerebrovascular accidents (CVA) like stroke or traumatic injury as revealed by documented experiments in that they could promote neovascularization and NBP and Ginkgo can reduce the infarct while berberine in addition to neuronal protection has an anti-infection component. NBP also appears to be able to limit CVA in hypertensive rats, suggesting an ability perhaps to control blood pressure. Apart from single agents, anti-infection components were also registered in multiformula of Bezoar chest functioning pill and Pien Tze Huang which might be useful in prevention and treatment of CNS condition.

To prevent potential degeneration of the nervous system, the ideal drug must be able (1) to promote circulation to the CNS, including inducing formation of new blood vessels upon ischemia; (2) to arrest or downregulate production and/or damage of superoxidants and free radicals, inclusive of NO; (3) to limit spread of lesion via reduction of edema, maintaining adequate ATP production, and preventing apoptosis or necrosis. Edema control in western medicine relied on diuretics and high osmotic sugar like mannitol. In Chinese medicine, some agents like natural musk (now becoming artificial musk) would prevent fluid accumulation. Although the above factors in research were certainly important and had been employed by many laboratories around the world as biomarkers for evaluating effects of Chinese herbal extracts or biomarkers on the nervous system, we in this laboratory regarded there should be further procedures to be performed as final evidence. This would be the evaluation of the function upon recovery or establishment of* de novo* collateral functioning utilizing upon recovery by physiological or imaging studies (functional MRI), in addition to merely employing behavior tests. An example of this was depicted by studies in our laboratory [38, 77–79]. It is also imperative to see how long the recovered function would last upon cessation of treatments, or from another angle, how long would the period of treatment had to be employed.

Apart from degeneration in the nervous system as a result of toxicity (e.g., by abusive drugs) during normal and abnormal aging, there were apparent cases of acute injury either as a result of traumatic injury or stroke. In such cases, particularly in those of stroke, herbal (either single or multiple in formulae) agents had been studied employing state-of-the-art scientific techniques in the past decade. These herbal agents have been proven useful in both pretreatment and posttreatment in animal models of stroke. During pretreatment, these drugs acted either to protect vessels of the nervous system or to control blood pressure whilst those for posttreatment aimed at limiting the lesion and preventing or reducing ischemic damages, as well as preventing radical and hyperoxide insults.

In addition to single drugs, there were two multidrug formulae well known for centuries which might have effects on CNS lesions, namely, Bezoar Chest functioning pill and Pien Tze Huang and the latter was originally introduced as an agent for liver disease. As the “Yang” of the liver upregulated in Chinese medicine, it led to CNS problems like stroke. Both of these drugs appeared to tackle the upregulation of “Yang” leading to CNS episodes. Out of these two formulae, only the latter had been evaluated for its action on the CNS via an evidence-based approach [80]. This drug essentially contained some similar components as those of the first drug, bezoar chest functioning pill, for example, both contained bile salt of bovine. This bile salt was widely used to downregulate the “heat” or “Yang” of the body. In the context of western medicine, it is probably an antibacterial agent. With the large amount of damaged tissues upon stroke, anti-infection would probably be a wise thing to initiate.

With the advance of herbal medicine to provide evidence for its action, researches in these subjects have reached an all-time high in the past decades. There were numerous projects comparing* in vivo* models with and without addition of a certain herbs or their constituents and thus to follow and evaluate the possible cellular involvement or its mechanism. These approaches, although valid, suffered from the inability to pinpoint whether the upregulation or downregulation of markers in these cells triggered by the drug was a specific or otherwise simple generalized effect. It is far better to derive controls by comparing western with herbal medication of similar therapeutic values rather than just delete the herb or its components as control. This more vigorous approach suggested here may be more convincing in the long way ahead to “modernize” herbal medicine and to compare and elucidate their real effects.

In closing, two traditionally used herbs and two recently discovered bioactive compounds derived from plants were reviewed here, with emphasis on similarities and differences in their CNS effects as well as supplementary information on other disease conditions. All of the herbs and compounds discussed possess antioxidant, antiapoptotic, and anti-Alzheimer's properties. Gingko and NBP may inhibit A*β*PP/A*β* pathway to contribute to cognitive improvement, whereas this is achieved by* Polygala* and berberine through increased ACh availability via cholinesterase inhibition. Both NBP and berberine also elicit anti-inflammatory effects. While recent findings advocate for the beneficial effects of NBP in other forms of dementia, potential values of berberine have been shown in affective disorders and diabetes. It is likely that herbs and plant-derived compounds, for example, berberine, have multiple sites of action and consequently are efficacious in a variety of disease conditions. New indications for existing and novel herbal extracts may be discovered with time.

## References

[1] Shi C, Yao Z, Xu J, Yew DT (2009). Effects of Gingko Extract (EGb761) on oxidative damage under different conditions of serum supply. *Journal of Bioenergetics and Biomembranes*.

[2] Shi C, Zhao L, Zhu B (2009). Dosage effects of EGb761 on hydrogen peroxide-induced cell death in SH-SY5Y cells. *Chemico-Biological Interactions*.

[3] Shi C, Xiao S, Liu J (2010). *Ginkgo biloba* extract EGb761 protects against aging-associated mitochondrial dysfunction in platelets and hippocampi of SAMP8 mice. *Platelets*.

[4] Shi C, Fang L, Yew DT, Yao Z, Xu J (2010). *Ginkgo biloba* extract EGb761 protects against mitochondrial dysfunction in platelets and hippocampi in ovariectomized rats. *Platelets*.

[5] Chandrasekaran K, Mehrabian Z, Spinnewyn B, Chinopoulos C, Drieu K, Fiskum G (2003). Neuroprotective effects of bilobalide, a component of *Ginkgo biloba* extract (EGb 761) in global brain ischemia and in excitotoxicity-induced neuronal death. *Pharmacopsychiatry*.

[6] Ahlemeyer B, Krieglstein J (2003). Neuroprotective effects of *Ginkgo biloba* extract. *Cellular and Molecular Life Sciences*.

[7] Shi C, Wu F, Yew DT, Xu J, Zhu Y (2010). Bilobalide prevents apoptosis through activation of the PI3K/Akt pathway in SH-SY5Y cells. *Apoptosis*.

[8] Shi C, Zhao L, Zhu B (2009). Protective effects of *Ginkgo biloba* extract (EGb761) and its constituents quercetin and ginkgolide B against *β*-amyloid peptide-induced toxicity in SH-SY5Y cells. *Chemico-Biological Interactions*.

[9] DeFeudis FV, Drieu K (2000). *Ginkgo biloba* extract (EGb 761) and CNS functions: basic studies and clinical applications. *Current Drug Targets*.

[10] Ahlemeyer B, Krieglstein J (2003). Pharmacological studies supporting the therapeutic use of *Ginkgo biloba* extract for Alzheimer’s disease. *Pharmacopsychiatry*.

[11] Loew D (2002). Value of *Ginkgo biloba* in treatment of Alzheimer dementia. *Wiener Medizinische Wochenschrift*.

[12] Mazza M, Capuano A, Bria P, Mazza S (2006). *Ginkgo biloba* and donepezil: a comparison in the treatment of Alzheimer’s dementia in a randomized placebo-controlled double-blind study. *European Journal of Neurology*.

[13] Schulz V (2003). Ginkgo extract or cholinesterase inhibitors in patients with dementia: what clinical trials and guidelines fail to consider. *Phytomedicine*.

[14] Wettstein A (2000). Cholinesterase inhibitors and Gingko extracts–are they comparable in the treatment of dementia? Comparison of published placebo-controlled efficacy studies of at least six months’ duration. *Phytomedicine*.

[15] Kanowski S, Hoerr R (2003). *Ginkgo biloba* extract EGb 761 in dementia: intent-to-treat analyses of a 24-week, multi-center, double-blind, placebo-controlled, randomized trial. *Pharmacopsychiatry*.

[16] Le Bars PL, Velasco FM, Ferguson JM, Dessain EC, Kieser M, Hoerr R (2002). Influence of the severity of cognitive impairment on the effect of the *Ginkgo biloba* extract EGb 761 in Alzheimer’s disease. *Neuropsychobiology*.

[17] Shi C, Liu J, Wu F, Yew DT (2010). *Ginkgo biloba* extract in Alzheimer’s disease: from action mechanisms to medical practice. *International Journal of Molecular Sciences*.

[18] Yew DT, Wong HW, Li WP, Lai HWL, Yu W-HA (1999). Nitric oxide synthase neurons in different areas of normal aged and Alzheimer’s brains. *Neuroscience*.

[19] Bastianetto S, Zheng W-H, Quirion R (2000). The *Ginkgo biloba* extract (EGb 761) protects and rescues hippocampal cells against nitric oxide-induced toxicity: involvement of its flavonoid constituents and protein kinase C. *Journal of Neurochemistry*.

[20] Liu P, Hu Y, Guo D-H (2010). Potential antidepressant properties of Radix Polygalae (Yuan Zhi). *Phytomedicine*.

[21] Lin Z, Gu J, Xiu J, Mi T, Dong J, Tiwari JK (2012). Traditional Chinese medicine for senile dementia. *Evidence-Based Complementary and Alternative Medicine*.

[22] Ikeya Y, Takeda S, Tunakawa M (2004). Cognitive improving and cerebral protective effects of acylated oligosaccharides in *Polygala tenuifolia*. *Biological and Pharmaceutical Bulletin*.

[23] Lee CI, Han JY, Hong JT, Oh KW (2013). 3, 4, 5-trimethoxycinnamic acid (TMCA), one of the constituents of Polygalae Radix enhances pentobarbital-induced sleeping behaviors via GABAAergic systems in mice. *Archives of Pharmacal Research*.

[24] Hu Y, Li J, Liu P (2012). Protection of SH-SY5Y neuronal cells from glutamate-induced apoptosis by 3,6 ′-disinapoyl sucrose, a bioactive compound isolated from radix polygala. *Journal of Biomedicine and Biotechnology*.

[25] Li C, Yang J, Yu S (2008). Triterpenoid saponins with neuroprotective effects from the roots of *Polygala tenuifolia*. *Planta Medica*.

[26] Kawashima K, Miyako D, Ishino Y, Makino T, Saito K-I, Kano Y (2004). Anti-stress effects of 3,4,5-trimethoxycinnamic acid, an active constituent of roots of *Polygala tenuifolia* (Onji). *Biological and Pharmaceutical Bulletin*.

[27] Zhu KY, Mao QQ, Ip SP (2012). A standardized chinese herbal decoction, kai-xin-san, restores decreased levels of neurotransmitters and neurotrophic factors in the brain of chronic stress-induced depressive rats. *Evidence-Based Complementary and Alternative Medicine*.

[28] Choi JG, Kim HG, Kim MC (2011). Polygalae radix inhibits toxin-induced neuronal death in the Parkinson’s disease models. *Journal of Ethnopharmacology*.

[29] Park H-J, Lee K, Heo H (2008). Effects of *Polygala tenuifolia* root extract on proliferation of neural stem cells in the hippocampal CA1 region. *Phytotherapy Research*.

[30] Zhang H, Han T, Zhang L (2008). Effects of tenuifolin extracted from radix polygalae on learning and memory: a behavioral and biochemical study on aged and amnesic mice. *Phytomedicine*.

[31] Sun X-L, Ito H, Masuoka T, Kamei C, Hatano T (2007). Effect of *Polygala tenuifolia* root extract on scopolamine-induced impairment of rat spatial cognition in an eight-arm radial maze task. *Biological and Pharmaceutical Bulletin*.

[32] Shin KY, Lee J-Y, Won BY (2009). BT-11 is effective for enhancing cognitive functions in the elderly humans. *Neuroscience Letters*.

[33] Yabe T, Iizuka S, Komatsu Y, Yamada H (1997). Enhancements of choline acetyltransferase activity and nerve growth factor secretion by Polygalae radix-extract containing active ingredients in Kami-untan-to. *Phytomedicine*.

[34] Li J, Zeng K-W, Shi S-P, Jiang Y, Tu P-F (2012). Anti-neuroinflammatory constituents from Polygala tricornis Gagnep. *Fitoterapia*.

[35] Park J-H, Kim JS, Jang DS, Lee S-M (2006). Effect of *Polygala tenuifolia* root extract on cerebral ischemia and reperfusion. *American Journal of Chinese Medicine*.

[36] Cheng M-C, Li C-Y, Ko H-C, Ko F-N, Lin Y-L, Wu T-S (2006). Antidepressant principles of the roots of *Polygala tenuifolia*. *Journal of Natural Products*.

[37] Limpert AS, Mattmann ME, Cosford ND (2013). Recent progress in the discovery of small molecules for the treatment of amyotrophic lateral sclerosis (ALS). *Beilstein Journal of Organic Chemistry*.

[38] Zhang L, Yu WH, Wang YX (2012). DL-3-n-Butylphthalide, an anti-oxidant agent, prevents neurological deficits and cerebral injury following stroke per functional analysis, magnetic resonance imaging and histological assessment. *Current Neurovascular Research*.

[39] Barone FC, Price WJ, White RF, Willette RN, Feuerstein GZ (1992). Genetic hypertension and increased susceptibility to cerebral ischemia. *Neuroscience and Biobehavioral Reviews*.

[40] Cui LY, Zhu YC, Gao S (2013). Ninety-day administration of dl-3-n-butylphthalide for acute ischemic stroke: a randomized, double-blind trial. *Chinese Medical Journal*.

[41] Zhang L, Lu L, Chan WM, Huang Y, Wai MSM, Yew DT (2012). Effects of DL-3-n-butylphthalide on vascular dementia and angiogenesis. *Neurochemical Research*.

[42] Lu X-L, Luo D, Yao X-L (2012). Dl-3n-butylphthalide promotes angiogenesis via the extracellular signal-regulated kinase 1/2 and phosphatidylinositol 3-kinase/akt-endothelial nitric oxide synthase signaling pathways. *Journal of Cardiovascular Pharmacology*.

[43] Xu J, Wang Y, Li N, Xu L, Yang H, Yang Z (2012). L-3-n-butylphthalide improves cognitive deficits in rats with chronic cerebral ischemia. *Neuropharmacology*.

[44] Wang HM, Zhang T, Huang JK, Sun XJ (2013). 3-N-butylphathalide (NBP) attenuates the amyloid-*β*-induced inflammatory responses in cultured astrocytes via the nuclear factor-kB signaling pathway. *Cellular Physiology and Biochemistry*.

[45] Feng X, Peng Y, Liu M, Cui L (2012). Dl-3-n-butylphthalide extends survival by attenuating glial activation in a mouse model of amyotrophic lateral sclerosis. *Neuropharmacology*.

[46] Peng Y, Hu Y, Xu S (2012). L-3-n-butylphthalide reduces tau phosphorylation and improves cognitive deficits in A*β*PP/PS1-Alzheimer’s transgenic mice. *Journal of Alzheimer’s Disease*.

[47] Peng Y, Sun J, Hon S (2010). L-3-n-butylphthalide improves cognitive impairment and reduces amyloid-*β* in a transgenic model of Alzheimer’s disease. *Journal of Neuroscience*.

[48] Wei W, Zhang W, Huang Y (2012). The therapeutic effect of (DL)-3-n-butylphthalide in rats with chronic cerebral hypoperfusion through downregulation of amyloid precursor protein and matrix metalloproteinase-2. *Journal of International Medical*.

[49] Feng XH, Yuan W, Peng Y, Liu MS, Cui LY (2012). Therapeutic effects of dl-3-n-butylphthalide in a transgenic mouse model of amyotrophic lateral sclerosis. *Chinese Medical Journal*.

[50] Zhao Q, Zhang C, Wang X, Chen L, Ji H, Zhang Y (2012). (S)-ZJM-289, a nitric oxide-releasing derivative of 3-n-butylphthalide, protects against ischemic neuronal injury by attenuating mitochondrial dysfunction and associated cell death. *Neurochemistry International*.

[51] Moghadam MH, Imenshahidi M, Mohajeri SA (2013). Antihypertensive efect of celery seed on rat blood pressure in chronic administration. *Journal of Medicinal Food*.

[52] Wang X, Wang L, Huang Z (2013). Synthesis and biological evaluation of nitric oxide releasing derivatives of 6-amino-3-n-butylphthalide as potential antiplatelet agents. *Bioorganic & Medicinal Chemistry Letters*.

[53] Singh IP, Mahajan S Berberine and its derivatives: a patent review (2009–2012). *Expert Opinion on Therapeutic Patents*.

[54] Ye M, Fu S, Pi R, He F (2009). Neuropharmacological and pharmacokinetic properties of berberine: a review of recent research. *Journal of Pharmacy and Pharmacology*.

[55] Han AM, Heo H, Kwon YK (2012). Berberine promotes axonal regeneration in injured nerves of the peripheral nervous system. *Journal of Medicinal Food*.

[56] Shigeta K, Ootaki K, Tatemoto H, Nakanishi T, Inada A, Muto N (2002). Potentiation of nerve growth factor-induced neurite outgrowth in PC12 cells by a coptidis rhizoma extract and protoberberine alkaloids. *Bioscience, Biotechnology and Biochemistry*.

[57] Chai YS, Yuan ZY, Lei F (2014). Inhibition of retinoblastoma mRNA degradation through poly-A-involved in the neuroprotective effect of berberine against cerebral ischemia. *PLoS ONE*.

[58] Lee T, Heo H, Kim Kwon Y (2010). Effect of berberine on cell survival in the developing rat brain damaged by MK-801. *Experimental Neurobiology*.

[59] Chen J-H, Huang S-M, Tan T-W (2012). Berberine induces heme oxygenase-1 up-regulation through phosphatidylinositol 3-kinase/AKT and NF-E2-related factor-2 signaling pathway in astrocytes. *International Immunopharmacology*.

[60] Luo T, Zhang H, Zhang W-W (2011). Neuroprotective effect of Jatrorrhizine on hydrogen peroxide-induced cell injury and its potential mechanisms in PC12 cells. *Neuroscience Letters*.

[61] Liu Y-Q, Cheng M-C, Wang L-X, Xiao H-B (2010). Rhizoma coptidis and berberine-induced activation of murine microglia N9 cells. *Journal of Ethnopharmacology*.

[62] Lu D-Y, Tang C-H, Chen Y-H, Wei I-H (2010). Berberine suppresses neuroinflammatory responses through AMP-activated protein kinase activation in BV-2 microglia. *Journal of Cellular Biochemistry*.

[63] Zhu F, Qian C (2006). Berberine chloride can ameliorate the spatial memory impairment and increase the expression of interleukin-1 beta and inducible nitric oxide synthase in the rat model of Alzheimer’s disease. *BMC Neuroscience*.

[64] Li H, Li XL, Zhang M (2014). Berberine ameliorates experimental autoimmune neuritis by suppressing both cellular and humoral immunity. *Scandinavian Journal of Immunology*.

[65] Singh DP, Chopra K (2013). Verapamil augments the neuroprotectant action of berberine in rat model of transient global cerebral ischemia. *European Journal of Pharmacology*.

[66] Bhutada P, Mundhada Y, Bansod K (2011). Protection of cholinergic and antioxidant system contributes to the effect of berberine ameliorating memory dysfunction in rat model of streptozotocin-induced diabetes. *Behavioural Brain Research*.

[67] Kim M, Cho KH, Shin MS (2014). Berberine prevents nigrostriatal dopaminergic neuronal loss and suppresses hippocampal apoptosis in mice with Parkinson's disease. *International Journal of Molecular Medicine*.

[68] Asai M, Iwata N, Yoshikawa A (2007). Berberine alters the processing of Alzheimer’s amyloid precursor protein to decrease A*β* secretion. *Biochemical and Biophysical Research Communications*.

[69] Kulkarni SK, Dhir A (2007). Possible involvement of l-arginine-nitric oxide (NO)-cyclic guanosine monophosphate (cGMP) signaling pathway in the antidepressant activity of berberine chloride. *European Journal of Pharmacology*.

[70] Kulkarni SK, Dhir A (2008). On the mechanism of antidepressant-like action of berberine chloride. *European Journal of Pharmacology*.

[71] Peng W-H, Lo K-L, Lee Y-H, Hung T-H, Lin Y-C (2007). Berberine produces antidepressant-like effects in the forced swim test and in the tail suspension test in mice. *Life Sciences*.

[72] Chu H, Jin G, Friedman E, Zhen X (2008). Recent development in studies of tetrahydroprotoberberines: mechanism in antinociception and drug addiction. *Cellular and Molecular Neurobiology*.

[73] Peng W-H, Wu C-R, Chen C-S, Chen C-F, Leu Z-C, Hsieh M-T (2004). Anxiolytic effect of berberine on exploratory activity of the mouse in two experimental anxiety models: interaction with drugs acting at 5-HT receptors. *Life Sciences*.

[74] Maes M (1995). Alterations in plasma prolyl endopeptidase activity in depression, mania, and schizophrenia: effects of antidepressants, mood stabilizers, and antipsychotic drugs. *Psychiatry Research*.

[75] Moghaddam HK, Baluchneijadmojarad T, Roghani M (2014). Berberine ameliorate oxidative stress and astrogliosis in the hippocampus of STZ-induced diabetic rats. *Molecular Neurobiology*.

[76] di Pierro F, Putignano P, Villanova N (2013). Preliminary study about the possible glycemic clinical advantage in using a fixed combination of Berberis aristata and Silybum mariaum standardized extracts versus only Berberis aristata in patients with type 2 diabetes. *Clinical Pharmacology*.

[77] Zhang L, Lam WP, Lu L (2011). How would composite traditional Chinese medicine protect the brain—an example of the composite formula “Pien Tze Huang”. *Current Medicinal Chemistry*.

[78] Wang C, Zheng D, Xu J, Lam W, Yew DT (2013). Brain damages in ketamine addicts as revealed by magnetic resonance imaging. *Frontiers in Neuroanatomy*.

[79] Sun L, Li Q, Zhang Y (2012). Chronic ketamine exposure induces permanent impairment of brain functions in adolescent cynomolgus monkeys. *Addiction Biology*.

[80] Zhang L, Lam WP, Lu L (2010). Protective effects and potential mechanisms of Pien Tze Huang on cerebral chronic ischemia and hypertensive stroke. *Chinese Medicine*.

